# Pattern of Cortical Fracture following Corticotomy for Distraction Osteogenesis

**DOI:** 10.5704/MOJ.1511.005

**Published:** 2015-11

**Authors:** M Luvan, SR Kanthan, G Roshan, A Saw

**Affiliations:** Department of Orthopaedic Surgery, Pulau Pinang Hospital, Georgetown, Malaysia; * Department of Orthopaedic Surgery, University of Malaya, Kuala Lumpur, Malaysia

**Keywords:** Distraction, Corticotomy, Osteogenesis, Lengthening

## Abstract

Corticotomy is an essential procedure for deformity correction and there are many techniques described. However there is no proper classification of the fracture pattern resulting from corticotomies to enable any studies to be conducted. We performed a retrospective study of corticotomy fracture patterns in 44 patients (34 tibias and 10 femurs) performed for various indications. We identified four distinct fracture patterns, Type I through IV classification based on the fracture propagation following percutaneous corticotomy. Type I transverse fracture, Type II transverse fracture with a winglet, Type III presence of butterfly fragment and Type IV fracture propagation to a fixation point. No significant correlation was noted between the fracture pattern and the underlying pathology or region of corticotomy.

## Introduction

Distraction osteogenesis is a procedure in which a bone is fractured and gradually distracted to allow correction of deformity or lengthening of the bone. Surgical procedure of fracturing the bone can be divided into two types, namely osteotomy and corticotomy^1,2^. Osteotomy involves dividing the bone through its entire width involving the periosteum, cortex, endosteum and the medullary canal. Corticotomy however involves dividing the bone through only its cortex with the preservation of the periosteal and endosteal layers. The procedure allows preservation of blood supply to the bone ends, and improves the quality of new bone formation^3^As first described by Gavriil Ilizarov the technique of corticotomy involves the use of an osteotome to divide the anterior, lateral and medial cortices, while the posterior cortex is broken by rotational force^4^. De Bastiani later improved on this technique with the use of multiple drill holes connected with an osteotome to complete the corticotomy^1^. Both techniques have been shown to yield similar results in terms of bone regenerative quality and time to union and consolidation^5^.

Corticotomy is usually performed percutaneously to minimise the degree of soft tissue disruption of the fracture ends. It is a technically demanding procedure and has a steep learning curve. It may be difficult to control the direction and extent of the cortical break. One of the common complications is comminution of the fracture ends that reflects a more extensive soft tissue injury while creating the cortical break. Another complication is longitudinal extension of the fracture line that may involve the bone fixation site, resulting in loss of bone fixation stability. When there is incomplete corticotomy, additional surgery will be needed to complete the procedure^6^.

Although less desirable fracture patterns are uncommon, additional procedure may be necessary before gradual distraction can be initiated. Undesirable fracture patterns may also affect the outcome. It will be useful to have a classification of corticotomy that can be used for accurate documentation, guiding the management, prognostication, and communication between clinicians. Commonly used classifications for diaphyseal fractures including the AO /AOA are not suitable, because we do not expect spiral fracture and segmental fracture following corticotomy^6^. We searched the English medical literature for classification of corticotomy but failed to find any. Therefore, we decided to conduct a study to review patterns of corticotomy and investigate its association with demographic factors and characteristics of the bone.

## Materials and Methods

We conducted a retrospective study of corticotomy procedures for either femur or tibia performed at our centre between January 2010 and December 2013. Indications for corticotomy included limb lengthening, angular correction and bone transport for bone defects. Only cases with complete pre-operative, immediate and delayed postoperative radiograph films were included. Further exclusions were made for cases where we could not find at least one post-operative radiograph that showed the whole extent of corticotomy site (without being blocked by components of external fixator). The first author reviewed all the radiographic films.

All the procedures were performed by senior orthopaedic surgeons with at least four years of experience in Orthopaedics and two or more years experience in Ilizarov surgeries. The technique used is the modified De’Bastiani procedure where a small incision measuring about 25 mm long is made over the site of proposed corticotomy and percutaneous minimal elevation of the periosteum was done. Following this, pre drilling was performed with a 3.2 mm drill bit (about 3 drill passes on the near cortex and about 4 to 5 passes on the far cortex). The corticotomy was then completed using a straight broad and narrow osteotome and mallet. Closure was done with one or two subcutaneous interrupted sutures with Vicrly 2/0 and two interrupted Nylon 3/0 suture for the skin.

We grouped the radiological findings according to geometry of the fracture line, extent of fracture and presence of comminution. We subsequently correlated the corticotomy patterns with the patients’ age, sex, involved bone, location of the corticotomy (proximal, middle or distal third) and the primary pathology (malunion, childhood infection and congenital/developmental deformities). These factors were considered as variables as they could potentially influence the compliance and support of the bone, which will affect the way the energy from the osteotome is dissipated through the bone. Comparison was done - using Chi-square test.

## Results

We recruited 44 patients who were treated for bone deformity or limb shortening during the study period. There were 29 males and 15 female patients with average age of 21 (range 5 years to 58 years). There were 34 patients with tibia and 10 with femur corticotomy. Out of this, 13 were proximal tibia, 17 midshaft tibia and four distal tibia. For the femur six were distal , three midshaft and one proximal. In some cases the whole extent of corticotomy could only be observed following some amount of lengthening. Based on the observations, we noted four patterns of the cortical fractures. Type I is a clean transverse corticotomy (Fig. 1A). Type II is a transverse corticotomy with a “winglet” / either a proximal or distal extension (Fig. 1B). Type III is a transverse corticotomy with a “butterfly” fragment / both proximal and distal extension (Fig. 1C). Type IV refers to any cortical fracture that extends into an adjacent fixation point, rendering the bone fixation unstable (Fig. 1D). A schematic drawing of the corticotomy patterns was made to show the different types of corticotomy based on the proposed classification (Fig. 2)

**Fig. 1a:**
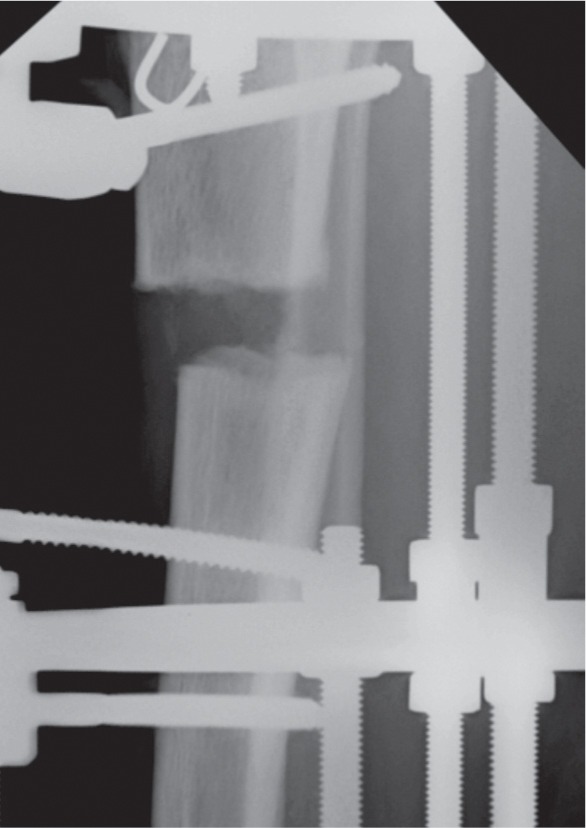
Radiograph showing Type I corticotomy with clean transverse cortical fracture.

**Fig. 1b:**
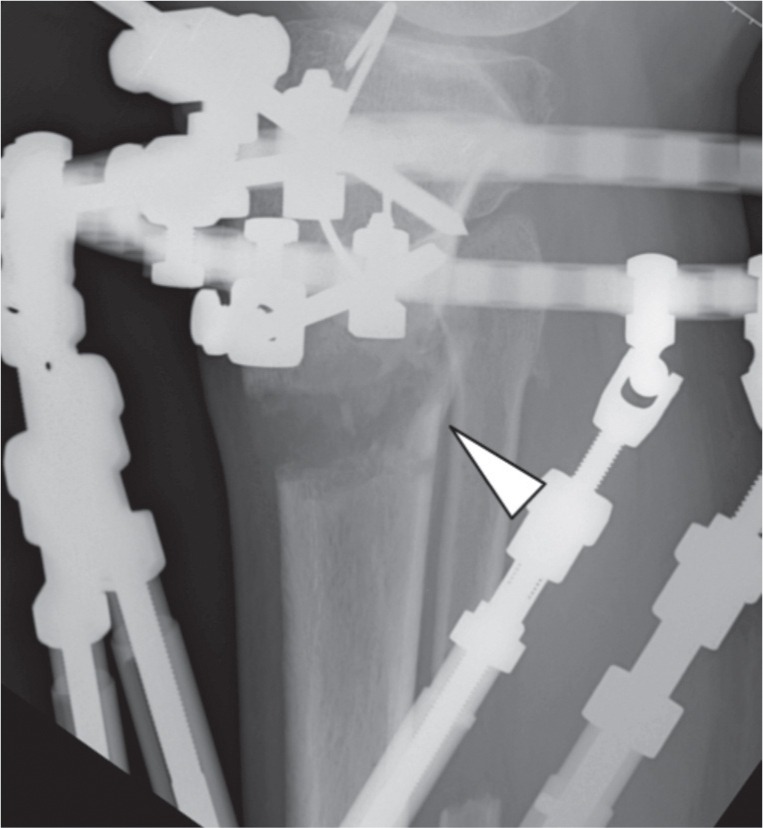
Radiograph showing Type II corticotomy with a “winglet”.

**Fig. 1c:**
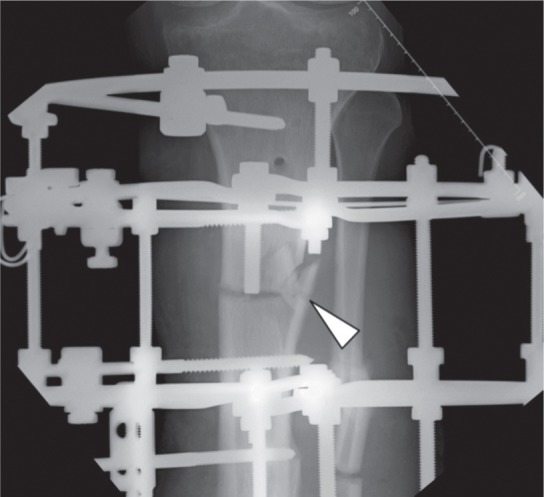
Radiograph showing Type III corticotomy with a “butterfly” fragment.

**Fig. 1d:**
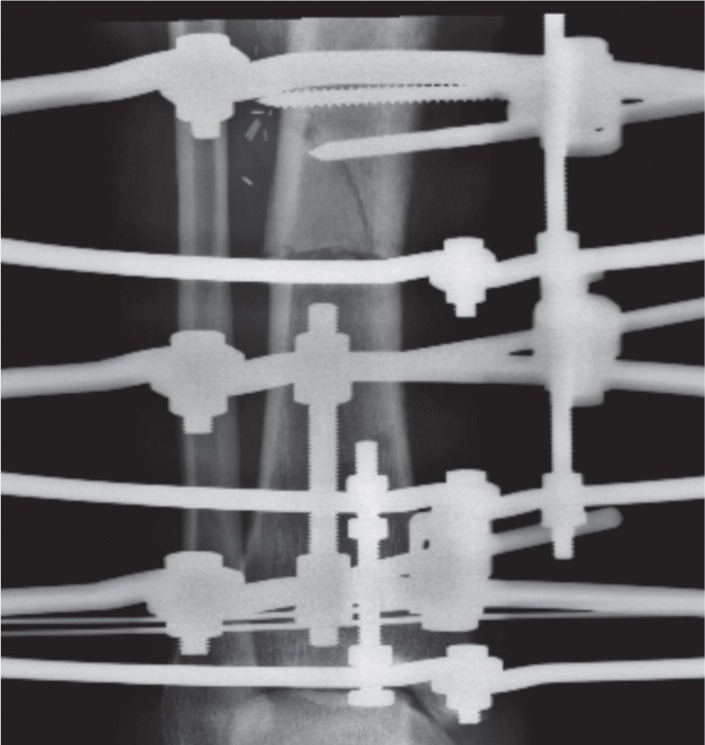
Radiograph showing Type VI corticotomy showing fracture extension into proximal half pin fixation.

**Fig. 2:**
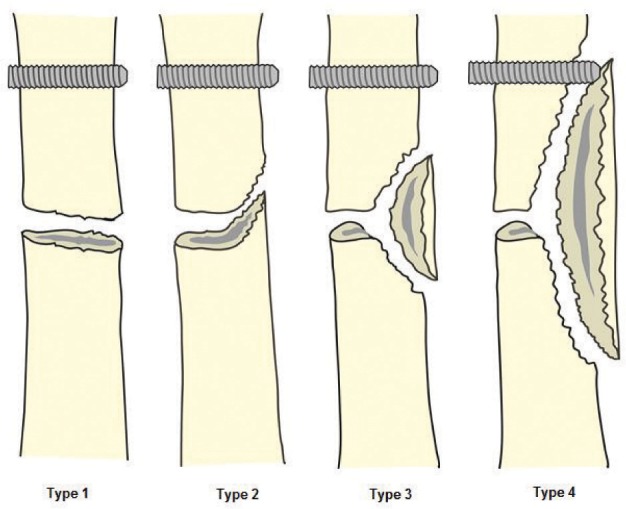
Figure 2 is a schematic drawing of the four - types of fracture patterns.

In our study, 31 patients had a type I corticotomy, forming the largest group. Two had a type II corticotomy, while the 10 had a type III corticotomy making up the second largest group. Only one patient among the 44 had a type IV corticotomy, where additional procedure was necessary to maintain fixation stability (Fig. 1D). None of the patients who had any of the other types of fracture needed additional procedures.

We further investigated several factors that may have influenced the pattern of the corticotomy (Table I). We correlated the patients’ age, sex, bone, location and underlying pathological cause to determine if there was a relationship with the types of cortical fractures. Due to the small sample size, we grouped Types I and II together (Group 1) as the corticotomy pattern was relatively similar, and compared them with Type III corticotomy (Group 2). Type IV was excluded from analysis as the sample size was too small to be statistically relevant. We were not able to find any statistically significance difference between the two groups of corticotomy patterns against these variables (Table II).

**Table I:** Frequency distribution of group 1 (Type 1 & 2) and group 2 (Type 3) corticotomy

**Group**	**Frequency**	**Percentage**
1 (Type 1 & 2)	33	76.7
2 (Type 3)	10	23.3
Total	43	100

**Table II:** Association between fracture patterns (group 1 and group 2) and gender, bone, pathology, age and bone segment

		**Group 1**	**Group 2**	**Chi-square value**	**p-value**
Gender	Male	6 (21.4%)	22 (78.6%)	0.150	0.698
	Female	4 (26.7%)	11(73.3%)		
Bone	Tibia	6 (18.2%)	27 (81.8%)		
	Femur	4 (40.0%)	6(60.0%)	2.047	0.153
	Total	10 (23.3%)	33 (76.7%)		
Pathology	Congenital Developmental	8 (22.9%)	27 (77.1%)	0.017	0.897
	Traumatic	2 (25.0%)	6 (75.0%)		
Age	Age ≥20	5 (27.8%)	13 (72.2%)	0.355	0.551
	Age <20	5 (20.0%)	20 (80.0%)		
Segment	Middle	6 (28.6%)	15 (71.4%)	0.977	0.613
	Distal	2 (25.0%)	6 (75.0%)		
	Proximal	2 (14.3%)	12 (85.7%)		

## Discussion

There have been no previous studies describing the fracture patterns of the corticotomy. We observed four patterns of cortical fracture in our series of corticotomy procedures. About 70 percent of our corticotomy ended up with a straight transverse cut and this is considered the most desirable pattern of corticotomy in terms of the quality of the regeneration and shortest consolidation time^3^. Gigli Saw technique is another method to perform corticotomy but there is conflicting evidence of its superiority compared to the modified De’ Bastiani technique^5,7^. None of the authors made any distinction of the fracture pattern. Type II corticotomy is essentially similar to Type I but there may be a higher risk of longitudinal extension of the fracture line. The second largest group was Type III corticotomy with comminution over the far cortex. This reflected higher energy involvement in the creation of the cortical break, which is undesirable in terms of bone healing. Type IV corticotomy that extends into the proposed fixation point is considered the worst outcome because this would weaken the stability of bone fixation. If the extension was not recognised, the resulting instability may adversely affect the bone healing and end up with complications like non-union or malunion^6^. Only one out of 44 corticotomy in our series showed Type 4 corticotomy, and we decided to perform additional fixation without removal of the involved half pin as we felt that it may still provide some degree of stability especially on axial loading.

We were not able to associate group 1 and group 2 corticotomy with any factors related to the patients or the bone. This may imply that risk factors that dictate the pattern of corticotomy are probably related to surgical technique. It is our impression that comminution on the far cortex and longitudinal extension of cortical fracture are related to inadequate drill holes on the opposite cortex or inadvertent change in the direction of the osteotome as it advances through the bone. We also felt that greater force may have been used to fracture the cortical bones, which was associated with greater tissue damage. However, we do not have adequate data to support these postulations.

The main limitation of our study was the small number of cases. This retrospective study was also not able to provide information on quality of bone healing following various types of corticotomy. We hope that this classification of corticotomy patterns will provide an essential variable for future studies to investigate factors influencing bone healing.

## Conclusion

Patterns of cortical fracture following corticotomy for bone lengthening can be grouped into four main types, clear transverse corticotomy (Type I corticotomy) was the most common pattern. We hope that this classification of corticotomy pattern will provide an essential variable for future studies to investigate factors influencing bone healing as we believe it reflects the amount of energy expended and the resulting fixation stability following corticotomy. Further studies are needed to assess the final outcome based on these fracture patterns.
